# Association of Electronic Cigarette Use by US Adolescents With Subsequent Persistent Cigarette Smoking

**DOI:** 10.1001/jamanetworkopen.2023.4885

**Published:** 2023-03-27

**Authors:** Ruoyan Sun, David Méndez, Kenneth E. Warner

**Affiliations:** 1 Department of Health Policy and Organization, School of Public Health, University of Alabama at Birmingham, Birmingham; 2 Department of Health Management and Policy, School of Public Health, University of Michigan, Ann Arbor

## Abstract

**Importance:**

Many studies have reported a positive association of youth electronic cigarette (e-cigarette) use with subsequent cigarette smoking initiation, but it remains unclear whether e-cigarette use is associated with continued cigarette smoking after initiation.

**Objective:**

To assess the association of youth baseline e-cigarette use with their continued cigarette smoking 2 years after initiation.

**Design, Setting, and Participants:**

The Population Assessment of Tobacco and Health (PATH) Study is a national longitudinal cohort study. This sample consisted of youth who participated in waves 3, 4, and 5 of the study (wave 3 was from October 2015 to October 2016, wave 4 was from December 2016 to January 2018, and wave 5 was from December 2018 to November 2019) and had never used cigarettes (cigarette-naive) by wave 3. The current analysis used multivariable logistic regressions in August 2022 to assess the association between e-cigarette use among cigarette-naive adolescents aged 12 to 17 years in 2015 and 2016 and subsequent continued cigarette smoking. PATH uses audio computer-assisted self-interviewing and computer-assisted personal interviewing to collect data.

**Exposures:**

Ever and current (past 30-day) use of e-cigarettes in wave 3.

**Main Outcomes and Measures:**

Continued cigarette smoking in wave 5 after initiating smoking in wave 4.

**Results:**

The current sample included 8671 adolescents who were cigarette naive in wave 3 and also participated in waves 4 and 5; 4823 of the participants (55.4%) were aged 12 to 14 years, 4454 (51.1%) were male, and 3763 (51.0%) were non-Hispanic White. Overall, regardless of e-cigarette use, few adolescents (362 adolescents [4.1%]) initiated cigarette smoking at wave 4, and even fewer (218 participants [2.5%]) continued smoking at wave 5. Controlling for multiple covariates, the adjusted odds ratio of baseline ever e-cigarette use, compared with never e-cigarette use, was 1.81 (95% CI, 1.03 to 3.18) for continued smoking measured as past 30-day smoking at wave 5. However, the adjusted risk difference (aRD) was small and not significant. The aRD was 0.88 percentage point (95% CI, −0.13 to 1.89 percentage points) for continued smoking, with the absolute risk being 1.19% (95% CI, 0.79% to 1.59%) for never e-cigarette users and 2.07% (95% CI, 1.01% to 3.13%) for ever e-cigarette users. Similar results were found using an alternative measure of continued smoking (lifetime ≥100 cigarettes and current smoking at wave 5) and using baseline current e-cigarette use as the exposure measure.

**Conclusions and Relevance:**

In this cohort study, absolute and relative measures of risks yielded findings suggesting very different interpretations of the association. Although there were statistically significant odds ratios of continued smoking comparing baseline e-cigarette users with nonusers, the minor risk differences between them, along with the small absolute risks, suggest that few adolescents are likely to continue smoking after initiation regardless of baseline e-cigarette use.

## Key Points

**Question:**

Among adolescents who initiate smoking after electronic cigarette (e-cigarette) use, is their baseline e-cigarette use status associated with continued smoking a few years later?

**Findings:**

This cohort study using data from a national sample of 8671 cigarette-naive adolescents found that youth who had used e-cigarettes at baseline, compared with those who had not, had higher odds of continuing smoking 2 years following initiating smoking the year after baseline. However, the absolute risks of continued smoking for both baseline e-cigarette users and nonusers were very small, as were the differences in absolute risks.

**Meaning:**

Although the significantly higher odds of continued smoking among e-cigarette users suggest a potentially important problem, the small magnitude of absolute risks and the minor risk differences in continued smoking between baseline e-cigarette users and nonusers indicate a much less consequential problem: few adolescents are likely to report continued smoking after initiation regardless of baseline e-cigarette use.

## Teaser

This cohort study assesses the association of youth baseline electronic cigarette use with continued cigarette smoking 2 years after initiation.

## Introduction

Since 2014, electronic cigarettes (e-cigarettes) have become the most popular nicotine or tobacco product among adolescents in the US.^1,2,3,4^ After the peak in 2019, the prevalence of current e-cigarette use (any use in the past 30 days) in high school students who completed the National Youth Tobacco Survey decreased to 14.1% in 2022.^2,3,4,5^

An important concern about the popularity of adolescent e-cigarette use is that it may lead cigarette-naive adolescents to try cigarettes. These adolescents could become addicted to nicotine and start smoking regularly, leading to well-known smoking-related health consequences in the future.^6^ On the basis of a recent meta-analysis,^7^ many longitudinal studies of adolescents in the US^8,9,10,11,12,13,14,15,16,17^ and other countries^18,19,20,21,22,23,24^ have reported positive associations between e-cigarette use and subsequent smoking initiation. However, all of these studies (except one^9^) examined only smoking initiation at follow-up, evaluating ever smoking of cigarettes. These studies provide no insight into whether these adolescents, after initiating smoking, become regular or established smokers.

Most adolescents who have ever tried smoking do not become regular or established smokers.^25^ Adolescent smoking is commonly conceptualized as progressing through a sequence of developmental states, defined by smoking frequency and intensity.^26^ Experimenters have tried a cigarette but do not smoke regularly.^27^ Established smokers are those who smoke regularly and have smoked at least 100 cigarettes during their lifetime.^28^ Data from the 2015 National Health Interview Survey indicate that among individuals aged 25 years who had ever tried a cigarette, only 36% reported lifetime use of more than 100 cigarettes.^29^ That number includes many who never smoked regularly or no longer do so.

It is important to distinguish experimenters from regular or established smokers. For adolescents who initiate smoking after e-cigarette use but do not continue smoking, their risks of adverse smoking-related health outcomes are negligible. However, if these adolescents become long-term smokers, then the health consequences could be substantial. Because most previous studies investigated only the association between e-cigarette use and subsequent smoking initiation,^8,10,11,12,13,14,15,16,17,18,19,20,21,22,23,24^ the association between e-cigarette use and continued smoking after initiation remains unclear.

To address this gap in the literature, we examined whether cigarette-naive adolescents who had used e-cigarettes at baseline, compared with those who had not used e-cigarettes, were more likely to continue smoking 2 years following initiating smoking the year after baseline. We used longitudinal data on adolescents from the 3 most recent waves (waves 3, 4, and 5) of the Population Assessment of Tobacco and Health (PATH) Study. We assessed baseline e-cigarette use in 2 ways: ever use and current use. On the basis of cigarette smoking status in waves 4 and 5, we then constructed 5 different measures of continued cigarette smoking. In assessing the prospective association between e-cigarette use and continued smoking, we controlled for multiple independent risk factors, including sociodemographics, environmental factors, other substance use, cigarette susceptibility, and mental health measures.

## Methods

### Data and Sample

The PATH Study is a US national longitudinal cohort study of youth and adults from the civilian, noninstitutionalized population. The survey uses a 4-stage, stratified probability sample design to select participants to answer questions on tobacco use and how it affects their health. The first wave of data collection took place in 2013, and annual or biennial follow-up surveys have been conducted since then. Follow-up surveys for individual participants were conducted during a target data collection period, starting with 1 month before the anniversary month of the individual’s prior survey and ending 1 month after the anniversary month. Adolescents were recruited for the PATH Study after written informed consent was given by their parents. The wave 3 weighted response rate was 83.3%.^30^ Our sample consisted of youth aged 12 to 17 years in 2015 and 2016 who participated in waves 3, 4, and 5 of the study (wave 3 was from October 2015 to October 2016, wave 4 was from December 2016 to January 2018, and wave 5 was from December 2018 to November 2019) and had never used cigarettes (cigarette-naive) by wave 3. We also included adolescents who turned 18 years old in wave 4 or 5 from the adult surveys. This study followed the Strengthening the Reporting of Observational Studies in Epidemiology (STROBE) reporting guidelines for cohort studies. The University of Alabama at Birmingham institutional review board exempted this study from review because it used deidentified data.

### Measures

The independent variable of interest is self-reported e-cigarette use in wave 3, assessed by 2 different measures: ever use and current (past 30-day) use. Cigarette smoking was assessed in waves 4 and 5, from both the youth and adult surveys. Among cigarette-naive adolescents in wave 3, their initiation of cigarette smoking was determined by self-reported ever use of cigarettes in wave 4. In wave 5, to assess continued cigarette use among wave 4 smoking initiators, we constructed 5 different binary measures of cigarette smoking representing, in order, progressively more substantial commitment to smoking: (1) any use in the past 12 months (continued smoking measure [CSM]–I); (2) any use in the past 30 days (CSM-II); (3) established use, which we defined as 100 cigarettes or more in the respondent’s lifetime and smoking currently (CSM-III); (4) 100 or more lifetime cigarettes and smoking 5 or more days in the past 30 days (CSM-IV); and (5) 100 or more lifetime cigarettes and smoking 20 or more days in the past 30 days (CSM-V).

### Study Covariates in Wave 3

Sociodemographic variables included age (12-14 vs 15-17 years), sex (male vs female), race and ethnicity (Hispanic, non-Hispanic Black, non-Hispanic White, and non-Hispanic other [ie, American Indian or Alaska Native, Asian Indian, Chinese, Filipino, Japanese, Korean, Vietnamese, other Asian, Native Hawaiian, Guamanian or Chamorro, Samoan, and other Pacific Islander]), highest parental education (high school or general educational development or less, some college, and college or higher), annual household income (<$50 000, $50 000-$100 000, and >$100 000), and school grades (less than mostly Bs vs mostly Bs and higher). All responses were self-reported. Race and ethnicity were analyzed in this study because they are factors associated with smoking.

Exposure to smoking was measured via family tobacco use, secondhand smoke exposure, and peer cigarette smoking. Family tobacco use (0 vs 1) was evaluated by asking whether anyone living with the respondent uses cigarettes, smokeless tobacco, cigars, cigarillos, filtered cigars, or any other form of tobacco. Secondhand smoke exposure (0 vs 1) was categorized by any nonzero answer to the question, “During the past seven days, about how many hours were you around others who were smoking?” Peer cigarette use (0 vs 1) was scored 1 if participants reported any positive number to the question asking, “How many of your best friends smoke cigarettes?”

Ever other tobacco product use (0 vs 1), excluding cigarettes and e-cigarettes, was defined by any positive response to questions asking about ever use of cigar, pipe, hookah, snus, smokeless tobacco, bidi, kretek, or dissolvable tobacco. Past 12-month use of alcohol (0 vs 1) and cannabis (0 vs 1) were assessed separately by the question, “In the past 12 months, have you used alcohol (or marijuana hash, THC, grass, pot or weed)?”

On the basis of previous literature,^31^ we constructed a binary variable of susceptibility to cigarette smoking. Participants were asked about potential future use of cigarettes: “Have you ever been curious about smoking a cigarette?”, “Do you think that you will try a cigarette soon?”, “If one of your best friends were to offer you a cigarette, would you smoke it?”, and “Do you think you will smoke a cigarette in the next year?” Participants answering “not at all curious” to the first question and “definitely not” to the last 3 were considered not susceptible to cigarettes, whereas the rest were considered susceptible.

We also included measures of internalizing and externalizing mental health problems, which are important factors associated with substance use.^32,33^ Respondents were asked the last time they experienced any of 4 internalizing disorder symptoms and any of 7 externalizing disorder symptoms. The number of reported symptoms within the past year was summed and then coded into a 3-level severity measure: low, moderate, and high.^34^ See eTable 1 in Supplement 1 for details.

### Statistical Analysis

The analyses were conducted in August 2022 using Stata statistical software version 17 (StataCorp), with the Fay method of balanced repeated replication to estimate variance. Stata’s svy command was used to incorporate survey weights. The income variable had the most missing data (6.6%), whereas others had few missing values (<5.0%). Because of the negligible impact of missing data,^35^ we conducted complete case analysis. We performed multivariable logistic regressions to examine the association between e-cigarette use in wave 3 and continued cigarette use 3 years later in wave 5, following smoking initiation in wave 4, controlling for the variables identified above.

We report our main results as adjusted odds ratios (aORs) as well as adjusted risk differences (aRDs) and absolute risks using Stata’s margins command. Two-tailed *P* < .05 by the Pearson χ^2^ test or Wald test was considered significant. An association that appears large according to ratios may be negligible when applied to the absolute risks and may, therefore, be misleading. As a result, Holmberg et al^36^ recommend reporting both the ratios and the actual risks.

In sensitivity analyses, we present the associations between e-cigarette use and continued cigarette use CSM-II and CSM-III in the main results and the associations with other continued use measures (CSM-I, CSM-IV, and CSM-V) as sensitivity checks. In addition to ever and current use of e-cigarettes at baseline, we add past 12-month e-cigarette use as a sensitivity check.

## Results

Among the 8671 adolescents included in the analysis, 4823 (55.4%) were aged 12 to 14 years, 4454 (51.1%) were male, and 3763 (51.0%) were non-Hispanic White (Table 1). A total of 842 participants (9.6%) reported ever e-cigarette use, and 138 (1.6%) reported current use. Highest parental education was high school or general educational development or less (3057 participants [31.7%]), some college (2700 participants [31.2%]), and college or higher (2733 participants [37.0%]). There were 3 categories for annual household income: less than $50 000 (3956 participants [43.7%]), between $50 000 and $100 000 (2082 participants [26.7%]), and more than $100 000 (2060 participants [29.7%]). A total of 2383 participants (25.7%) reported school grades less than mostly Bs, and 6228 (74.3%) reported mostly Bs or higher. In total, 2535 participants (29.1%) lived with family members who used tobacco, 2393 (28.2%) were exposed to secondhand smoke, and 1403 (15.7%) had best friends who smoked cigarettes. A small fraction (438 participants [5.3%]) had ever used other tobacco products (excluding cigarettes and e-cigarettes) and used cannabis (207 participants [2.5%]) in the past 12 months, but 1508 (18.9%) reported past 12-month alcohol use. Less than one-third (2575 participants [29.6%]) were susceptible to cigarettes. Approximately one-half (4393 participants [50.4%]) had a low score for internalizing problems, and 3792 (42.9%) had a low score for externalizing problems. We found significant differences in almost all of these characteristics (excluding sex) between ever and never baseline e-cigarettes users. Similarly, most of these characteristics differed significantly between current e-cigarette users and non–current users.

**Table 1.** Sample Characteristics of Baseline (Population Assessment of Tobacco and Health Wave 3) Cigarette-Naive Adolescents, With Prevalence of Ever and Current e-Cigarette Use

Characteristics	Wave 3 never cigarette users (n = 8671)		Ever e-cigarette user (n = 842)			Current e-cigarette user (n = 138)		
	Participants, No.	Weighted, % (95% CI)	Participants, No.	Weighted, % (95% CI)	*P* value^a^	Participants, No.	Weighted,% (95% CI)	*P* value^a^
Age, y								
12-14	4823	55.4 (54.8-56.1)	231	4.8 (4.2-5.4)	<.001	36	0.8 (0.5-1.1)	<.001
15-17	3848	44.6 (43.9-45.3)	611	15.7 (14.6-16.9)		102	2.7 (2.2-3.3)	
Sex								
Male	4454	51.1 (50.5-51.8)	454	10.0 (9.1-11.1)	.24	74	1.8 (1.4-2.2)	.30
Female	4195	48.9 (48.3-49.5)	386	9.3 (8.5-10.1)		64	1.5 (1.2-1.9)	
Race and ethnicity								
Hispanic	2606	24.5 (23.9-25.2)	307	11.7 (10.4-13.2)	.01	40	1.5 (1.1-2.1)	.40
Non-Hispanic								
Black	1199	14.2 (13.6-14.7)	114	10.6 (8.9-12.5)		18	1.7 (1.1-2.8)	
White	3763	51.0 (50.3-51.8)	330	8.9 (7.9-10.0)		64	1.8 (1.4-2.3)	
Other^b^	792	10.3 (9.8-10.8)	75	8.6 (6.9-10.8)		12	1.0 (0.6-1.8)	
Highest parental education								
High school or general educational development or less	3057	31.7 (30.0-33.5)	341	11.2 (10.1-12.5)	<.001	50	1.7 (1.3-2.3)	.62
Some college	2700	31.2 (29.6-32.9)	288	10.9 (9.8-12.2)		44	1.8 (1.3-2.5)	
College or higher	2733	37.0 (34.9-39.3)	199	7.4 (6.3-8.6)		42	1.5 (1.1-2.0)	
Annual household income, $								
<50 000	3956	43.7 (41.8-45.5)	419	10.9 (9.9-11.9)	.02	57	1.5 (1.2-2.1)	.77
50 000-100 000	2082	26.7 (25.2-28.1)	202	9.3 (8.0-10.8)		35	1.7 (1.2-2.4)	
>100 000	2060	29.7 (27.5-32.0)	169	8.3 (6.9-9.8)		39	1.8 (1.2-2.7)	
School grades								
Less than mostly Bs	2383	25.7 (24.5-27.0)	335	14.3 (12.8-15.9)	<.001	51	2.3 (1.7-3.1)	.02
Mostly Bs and higher	6228	74.3 (73.0-75.5)	501	8.0 (7.3-8.8)		85	1.4 (1.1-1.8)	
Family tobacco use								
Yes	2535	29.1 (27.6-30.6)	343	13.9 (12.3-15.6)	<.001	56	2.4 (1.8-3.1)	<.001
No	6004	71.0 (69.4-72.5)	487	7.9 (7.3-8.7)		80	1.3 (1.1-1.7)	
Secondhand smoke								
Yes	2393	28.2 (26.9-29.6)	370	15.6 (14.0-17.3)	<.001	73	3.2 (2.5-4.1)	<.001
No	6056	71.8 (70.4-73.1)	440	7.1 (6.6-7.8)		59	1.0 (0.8-1.3)	
Peer cigarette use								
Yes	1403	15.7 (14.7-16.7)	273	20.1 (17.8-22.6)	<.001	41	3.0 (2.2-4.1)	<.001
No	7216	84.3 (83.3-85.3)	565	7.7 (7.1-8.3)		95	1.4 (1.1-1.7)	
Ever used other tobacco products^c^								
Yes	438	5.3 (4.9-5.9)	214	49.7 (45.4-54.1)	<.001	44	10.9 (8.3-14.2)	<.001
No	7757	94.7 (94.1-95.2)	577	7.3 (6.7-8.0)		87	1.1 (0.9-1.4)	
Used alcohol in past 12 mo								
Yes	1508	18.9 (17.8-20.1)	365	23.5 (21.2-26.0)	<.001	65	4.3 (3.4-5.6)	<.001
No	6889	81.1 (79.9-82.2)	462	6.6 (6.0-7.3)		70	1.0 (0.8-1.3)	
Used cannabis in past 12 mo								
Yes	207	2.5 (2.2-2.9)	85	41.9 (34.7-50.0)	<.001	15	6.9 (4.0-11.6)	<.001
No	8169	97.5 (97.1-97.8)	608	7.3 (6.8-8.0)		88	1.1 (0.9-1.4)	
Susceptible to cigarettes								
Yes	2575	29.6 (28.5-30.7)	435	16.8 (15.3-18.5)	<.001	87	3.5 (2.7-4.4)	<.001
No	6068	70.4 (69.3-71.5)	404	6.6 (6.0-7.2)		51	0.9 (0.7-1.2)	
Internalizing problems^d^								
Low	4393	50.4 (49.1-51.7)	358	8.1 (7.3-9.0)	<.001	60	1.4 (1.1-1.8)	<.001
Moderate	2398	28.1 (27.1-29.1)	220	9.1 (8.0-10.4)		25	1.1 (0.8-1.7)	
High	1880	21.6 (20.5-22.7)	264	13.9 (12.1-15.9)		53	2.9 (2.2-3.9)	
Externalizing problems^d^								
Low	3792	42.9 (41.6-44.1)	285	7.5 (6.6-8.5)	<.001	47	1.2 (0.9-1.7)	<.001
Moderate	2424	28.4 (27.4-29.4)	232	9.3 (8.1-10.7)		28	1.3 (0.8-1.9)	
High	2455	28.8 (27.6-30.0)	325	13.1 (11.6-14.7)		63	2.6 (2.0-3.3)	

^a^ Pearson χ^2^ test was performed to compare the prevalence of e-cigarette use across different groups of sample characteristics at wave 3.

^b^ Non-Hispanic other includes American Indian or Alaska Native, Asian Indian, Chinese, Filipino, Japanese, Korean, Vietnamese, other Asian, Native Hawaiian, Guamanian or Chamorro, Samoan, and other Pacific Islander.

^c^ Other tobacco products include cigar, pipe, hookah, snus, smokeless tobacco, bidi, kretek, and dissolvable tobacco.

^d^ Assessed by the Global Appraisal of Individual Needs–Short Screener.^34^ Adolescents were classified as low (0-1 symptom), moderate (2-3 symptoms), or high (≥4 symptoms). See eTable 1 in Supplement 1 for details.

In Table 2, we show the prevalence of cigarette initiation at wave 4 and continued use in wave 5. Overall, few adolescents started smoking cigarettes in wave 4, and even fewer continued smoking in wave 5. A total of 362 cigarette-naive adolescents in wave 3 (4.1% of all cigarette-naive adolescents) initiated cigarette use in wave 4. Among them, 218 (2.5%) continued smoking cigarettes using CSM-I, 133 (1.5%) using CSM-II, 60 (0.8%) using CSM-III, 27 (0.4%) using CSM-IV, and 12 (0.2%) using CSM-V.

**Table 2.** Prevalence of Cigarette Initiation at Population Assessment of Tobacco and Health Wave 4 and Continued Cigarette Use at Wave 5 Among Baseline Cigarette-Naive Adolescents Who Smoked at Wave 4, Using Various Definitions of Continued Use

Cigarette use	Definition	Participants, No. (N = 8671)	Weighted, % (95% CI)
Cigarette initiation	Past 12-mo use at wave 4	362	4.1 (3.7-4.6)
Continued smoking measure			
I	Past 12-mo use at wave 4 and past 12-mo use at wave 5	218	2.5 (2.2-2.9)
II	Past 12-mo use at wave 4 and past 30-d use at wave 5	133	1.5 (1.3-1.8)
III	Past 12-mo use at wave 4 and established use at wave 5^a^	60	0.8 (0.6-1.0)
IV	Past 12-mo use at wave 4, established use and ≥5 d use in the past 30 d at wave 5	27	0.4 (0.2-0.6)
V	Past 12-mo use at wave 4, established use and ≥20 d use in the past 30 d at wave 5	12	0.2 (0.0-0.3)

^a^ Established use was defined as lifetime of 100 cigarettes or more and currently smoking cigarettes.

Table 3 presents the prevalence of 2 of the continued cigarette use measures (CSM-II and CSM-III) by sample characteristics. Using CSM-II, among adolescents who had ever used e-cigarettes in wave 3, 6.0% (95% CI, 4.5%-8.0%) initiated smoking cigarettes in wave 4 and continued smoking in wave 5, compared with 1.1% (95% CI, 0.8%-1.3%) among never e-cigarette users. Using CSM-III, 3.6% (95% CI, 2.5%-5.1%) of ever e-cigarette users continued smoking, compared with 0.5% (95% CI, 0.3%-0.7%) of never e-cigarette users. Similarly, continued smoking prevalence in wave 5 was higher among current e-cigarette users in wave 3. On the basis of continued use measures CSM-II and CSM-III, 9.4% (95% CI, 5.1%-16.8%) and 7.4% (95% CI, 3.6%-14.6%) of current e-cigarette users in wave 3 continued smoking, respectively, compared with 1.4% (95% CI, 1.2%-1.7%) and 0.7% (0.3%-0.9%) of non–current users. In addition, age, race and ethnicity, school grades, family tobacco use, secondhand smoke, peer cigarette use, ever use of other tobacco products, past 12-month use of alcohol and cannabis, cigarette susceptibility, and scores for internalizing and externalizing problems were all factors significantly associated with both continued use measures.

**Table 3.** Continued Cigarette Use at Population Assessment of Tobacco and Health Wave 5 Among Baseline Cigarette-Naive Adolescents Who Initiated Smoking 1 Year Later, by Sample Characteristics at Wave 3

Wave 3 never cigarette users	Continued cigarette use			
	CSM-II (n = 133)^a^		CSM-III (n = 60)^b^	
	Weighted, % (95% CI)	*P* value^c^	Weighted, % (95% CI)	*P* value^c^
Ever used e-cigarettes				
Yes	6.0 (4.5-8.0)	<.001	3.6 (2.5-5.1)	<.001
No	1.1 (0.8-1.3)		0.5 (0.3-0.7)	
Used e-cigarettes in past 30 d				
Yes	9.4 (5.1-16.8)	<.001	7.4 (3.6-14.6)	<.001
No	1.4 (1.2-1.7)		0.7 (0.5-0.9)	
Age, y				
12-14	0.7 (0.5-1.0)	<.001	0.3 (0.2-0.5)	<.001
15-17	2.6 (2.1-3.1)		1.4 (1.0-1.8)	
Sex				
Male	1.6 (1.2-2.0)	.79	0.8 (0.6-1.2)	.54
Female	1.5 (1.1-2.0)		0.7 (0.5-1.1)	
Race and ethnicity				
Hispanic	1.6 (1.2-2.2)	.002	0.5 (0.3-0.8)	.01
Non-Hispanic				
Black	0.4 (0.2-0.9)		0.1 (0.0-0.5)	
White	2.0 (1.5-2.6)		1.1 (0.8-1.5)	
Other^d^	1.0 (0.5-2.1)		0.8 (0.3-1.9)	
Highest parental education				
High school or general educational development or less	1.7 (1.2-2.3)	.03	0.9 (0.6-1.3)	.32
Some college	2.0 (1.5-2.6)		0.9 (0.6-1.4)	
College or higher	1.1 (0.8-1.5)		0.6 (0.3-1.0)	
Annual household income, $				
<50 000	1.7 (1.3-2.2)	.42	0.8 (0.5-1.1)	.94
50 000-100 000	1.7 (1.2-2.4)		0.8 (0.5-1.4)	
>100 000	1.3 (0.9-1.8)		0.8 (0.5-1.3)	
School grades				
Less than mostly Bs	2.4 (1.8-3.2)	<.001	1.2 (0.8-1.8)	.01
Mostly Bs or higher	1.2 (1.0-1.5)		0.6 (0.4-0.9)	
Family tobacco use				
Yes	2.7 (2.0-3.6)	<.001	1.6 (1.1-2.3)	<.001
No	1.0 (0.8-1.3)		0.4 (0.3-0.6)	
Secondhand smoke				
Yes	3.3 (2.5-4.2)	<.001	1.7 (1.2-2.4)	<.001
No	0.8 (0.6-1.1)		0.4 (0.2-0.6)	
Peer cigarette use				
Yes	4.2 (3.3-5.4)	<.001	2.2 (1.5-3.2)	<.001
No	1.0 (0.8-1.3)		0.5 (0.3-0.7)	
Ever used other tobacco products^e^				
Yes	6.3 (4.1-9.5)	<.001	3.6 (2.0-6.3)	<.001
No	1.2 (1.0-1.5)		0.6 (0.4-0.8)	
Used alcohol in past 12 mo				
Yes	3.7 (2.8-4.8)	<.001	2.0 (1.3-3.1)	<.001
No	1.0 (0.8-1.4)		0.5 (0.3-0.7)	
Used cannabis in past 12 mo				
Yes	5.2 (2.7-10.0)	<.001	3.2 (1.3-7.8)	<.001
No	1.2 (1.0-1.5)		0.6 (0.4-0.8)	
Susceptible to cigarettes				
Yes	3.7 (3.0-4.5)	<.001	1.9 (1.4-2.6)	<.001
No	0.6 (0.4-0.8)		0.3 (0.1-0.5)	
Internalizing problems^f^				
Low	1.0 (0.8-1.4)	<.001	0.4 (0.3-0.7)	<.001
Moderate	1.4 (0.9-2.0)		0.5 (0.2-0.9)	
High	2.9 (2.2-3.9)		1.9 (1.4-2.7)	
Externalizing problems^f^				
Low	1.0 (0.7-1.4)	<.001	0.3 (0.2-0.6)	<.001
Moderate	1.5 (1.0-2.2)		0.9 (0.5-1.4)	
High	2.4 (1.9-3.0)		1.3 (0.9-1.9)	

Abbreviation: CSM, continued smoking measure.

^a^ Refers to past 12-month use at wave 4 and past 30-day use at wave 5.

^b^ Refers to past 12-month use at wave 4 and established use at wave 5. Established use is defined as lifetime of 100 cigarettes or more and currently smoking.

^c^ Pearson χ^2^ test was performed to compare the distribution of sample characteristics by continued cigarette use status at wave 5.

^d^ Non-Hispanic other includes American Indian or Alaska Native, Asian Indian, Chinese, Filipino, Japanese, Korean, Vietnamese, other Asian, Native Hawaiian, Guamanian or Chamorro, Samoan, and other Pacific Islander.

^e^ Other tobacco products include cigar, pipe, hookah, snus, smokeless tobacco, bidi, kretek, and dissolvable tobacco.

^f^ Assessed by the Global Appraisal of Individual Needs–Short Screener.^34^ Adolescents were classified as low (0-1 symptom), moderate (2-3 symptoms), or high (≥4 symptoms). See eTable 1 in Supplement 1 for details.

Table 4 shows the association of baseline e-cigarette use and 2 of our continued cigarette smoking measures (CSM-II and CSM-III), adjusting for all study covariates. With CSM-II, baseline ever e-cigarette use was associated with a 0.88 percentage point increase (95% CI, −0.13 to 1.89 percentage points) in reported continued smoking, from 1.19% (95% CI, 0.79% to 1.59%) for never e-cigarette users to 2.07% (95% CI, 1.01% to 3.13%) for ever e-cigarette users. Baseline current e-cigarette use was associated with a 1.88 percentage points increase (95% CI, −0.66 to 4.41 percentage points) in continued smoking, from 1.30% (95% CI, 0.90% to 1.70%) among noncurrent e-cigarette users to 3.18% (95% CI, 0.57% to 5.79%) among current users. The aRDs of using CSM-III were 0.60 percentage point (95% CI, −0.18 to 1.38 percentage points) for ever e-cigarette use and 1.79 percentage points (95% CI, −0.51 to 4.09 percentage points) for current use. Note that none of the aRDs was statistically significant. On the other hand, all aORs were statistically significant. The aORs were 1.81 (95% CI, 1.03 to 3.18) and 2.66 (95% CI, 1.07 to 6.63), respectively, for ever and current use of e-cigarettes with CSM-II. The aORs were 2.24 (95% CI, 1.06 to 4.72) and 4.59 (95% CI, 1.39 to 15.16), respectively, for ever and current use of e-cigarettes with CSM-III.

**Table 4.** Association Between Baseline e-Cigarette Use and Continued Cigarette Use in 3 Years, After Cigarette Initiation a Year After the Baseline

Wave 3 e-cigarette use	Wave 5 continued cigarette use			
	aOR (95% CI)^a^	aRD (95% CI)^b^	Risk without e-cigarette use, % (95% CI)^c^	Risk with e-cigarette use, % (95% CI)^c^
CSM-II^d^				
Ever use	1.81 (1.03 to 3.18)^e^	0.88 (−0.13 to 1.89)	1.19 (0.79 to 1.59)	2.07 (1.01 to 3.13)
Current use	2.66 (1.07 to 6.63)^e^	1.88 (−0.66 to 4.41)	1.30 (0.90 to 1.70)	3.18 (0.57 to 5.79)
CSM-III^f^^,^^g^				
Ever use	2.24 (1.06 to 4.72)^e^	0.60 (−0.18 to 1.38)	0.53 (0.19 to 0.88)	1.14 (0.21 to 2.07)
Current use	4.59 (1.39 to 15.16)^e^	1.79 (−0.51 to 4.09)	0.60 (0.23 to 0.97)	2.39 (0.01 to 4.76)

Abbreviations: aOR, adjusted odds ratio; aRD, adjusted risk difference; CSM, continued smoking measure.

^a^ aORs were adjusted for all study covariates: age, sex, race and ethnicity, highest parental education, household income, school grades, family tobacco use, secondhand smoke, peer cigarette use, ever other tobacco product use, past 12-month alcohol use, past 12-month cannabis use, cigarette susceptibility, internalizing problems, and externalizing problems.

^b^ aRD, in percentage points, was calculated as risk with e-cigarette use minus risk without e-cigarette use. Values were adjusted for all study covariates.

^c^ Denotes estimated risks for continued cigarette use in 3 years given baseline e-cigarette use status.

^d^ Refers to past 12-month use at wave 4 and past 30-day use at wave 5.

^e^ Statistically significant at *P* < .05.

^f^ Refers to past 12-month use at wave 4 and established use at wave 5. Established use is defined as lifetime of 100 cigarettes or more and currently smoking.

^g^ Because of the limited number of non-Hispanic Black participants who reported continued use (past 12-month use at wave 4 and established use at wave 5), we replaced the categorical measure of race (non-Hispanic White, non-Hispanic Black, Hispanic, and non-Hispanic other) with a binary measure (non-Hispanic White vs other).

We present the complete regression results of our main analyses in eTables 2 and 3 in Supplement 1. The results of sensitivity analyses are similar to those in Table 4. See eTables 4 and 5 in Supplement 1. Note that when using CSM-IV and CSM-V as the outcome, some findings were not significant because of the few participants reporting continued use. We also calculated associations between e-cigarette use and continued cigarette use without survey weights (eTable 6 in Supplement 1), and the results were very similar.

## Discussion

In general, in this cohort study, regardless of e-cigarette use status, few cigarette-naive adolescents (4.1%) initiated cigarette smoking and even fewer (≤2.5%) continued to smoke at all in 3 years. As seen in Table 2, the progressively more rigorous definitions of continued smoking (CSM-I to CSM-V) yielded successively lower prevalence in PATH wave 5, decreasing to 0.2% for CSM-V, more than 100 lifetime cigarettes and smoking 20 or more days in the past 30 days. Note that even our most demanding measure of regular smoking included many respondents who smoked less than every day.

According to the aORs in our analysis, we found that cigarette-naive adolescents who had used e-cigarettes at baseline, compared with those who had not, had significantly higher odds of continuing smoking 2 years after their initial smoking. In contrast, however, the aRDs were not significant, although baseline e-cigarette use was still associated with increases in risks of continued smoking. The magnitude of the risk difference was also very small, as were the absolute risks of continued smoking.

These seemingly conflicting findings are both accurate. Holmberg et al^36^ have observed that measures of ratios could change substantially with outcome prevalence, whereas measures of difference stay the same. In our study of continued smoking, because of the low outcome prevalence (1.5% with CSM-II and 0.6% with CSM-III), the observed small difference in absolute risks leads to a large value in terms of ratios. The association can be statistically significant and large using ratio measures without having a meaningful size of change of the outcome measure. Similarly, because relatively few adolescents initiate cigarette smoking in general, studies investigating the association of e-cigarettes with cigarettes that only report their findings as ratios may have exaggerated the importance of the association in the minds of readers. To fully and accurately describe this relationship, future studies should report absolute risks, as well as relative measures in ratios.

Although a positive association between e-cigarette use and cigarette initiation has been reported by many studies, at the population level, the prevalence of past 30-day cigarette smoking among youth and young adults has steadily decreased in the era of e-cigarettes.^37^ This apparent inconsistency between individual-level and population-level results can be explained by our findings. On the one hand, there is a positive association between e-cigarette use and subsequent smoking in terms of aORs. On the other hand, given the low prevalence of smoking, the absolute risks of continued smoking for both e-cigarette users and nonusers and the risk differences between them are very small. The change in the prevalence of cigarette smoking due to e-cigarette use at the population level is, thus, also very small.

The important question is whether e-cigarette use may lead substantial proportions of cigarette-naive adolescents to become long-term smokers. Consistent with previous findings showing that few youth experimenters become regular or established smokers,^25,29^ our results show that many adolescents who initiated smoking did not report continued smoking 2 years later. Considering our CSMs (CSM-II to CSM-V), the vast majority did not. In addition, a recent study by Méndez et al^38^ revealed that the adult smoking cessation rate in the US has continued to increase, from 4.2% in 2008 to 2013 to 5.4% to 2014 to 2019. This large increase exceeded the rate expected on the basis of the trend over time.^38^ The cessation rate has become especially large for young adults, and the smoking initiation rate has decreased greatly during the same period. As a result, it is likely that smoking prevalence, especially among young adults, will decline further in the coming years.

### Limitations

This study has a few limitations. First, our results using data from PATH waves 3 through 5 (2015-2019) may not hold for data from other years. One possible reason is changes in e-cigarette prevalence. Youth e-cigarette use increased between 2017 and 2019 and then decreased after 2019. Future studies are needed to examine whether the association between e-cigarette use and continued cigarette smoking changes over time. Another limitation is with respect to the study period of continued smoking. We only assessed continued smoking 3 years after the baseline survey and 2 years after smoking initiation. It is possible that the prevalence of continued smoking could change if we used a longer study period. Future analysis can be conducted once PATH wave 6 data become available. Furthermore, there is the possibility of response bias, which is a common problem with self-reported data. However, Bachman et al^39^ found evidence supporting the validity of self-reported data.

## Conclusions

In this cohort study of cigarette-naive respondents, we examined the association of adolescent e-cigarette use at baseline with continued cigarette smoking 3 years later, following smoking initiation a year after baseline. Our findings suggest very different interpretations of the association as measured by absolute and relative risk. The minor risk differences in continued smoking among baseline e-cigarette users and nonusers, together with the small magnitude of absolute risks for both groups, suggest that regardless of baseline e-cigarette use, few adolescents were likely to report continued smoking after initiation.

## Notes

Supplement 1. **eTable 1.** Global Appraisal of Individual Needs–Short Screener (GAIN-SS) Items **eTable 2. **Association Between Baseline e-Cigarette Use and Continued Cigarette Use (Continued Smoking Measure II) in 3 Years, After Cigarette Initiation a Year After the Baseline **eTable 3.** Association Between Baseline e-Cigarette Use and Continued Cigarette Use (Continued Smoking Measure III) in 3 Years, After Cigarette Initiation a Year After the Baseline **eTable 4.** Association Between Baseline e-Cigarette Use and Continued Cigarette Use in 3 Years, After Cigarette Initiation a Year After the Baseline, With Alternative Measures of Continued Use **eTable 5.** Association Between Baseline Past 12-Month e-Cigarette Use and Continued Cigarette Use in 3 Years, After Cigarette Initiation a Year After the Baseline **eTable 6.** Association Between Baseline e-Cigarette Use and Continued Cigarette Use in 3 Years, After Cigarette Initiation a Year After the Baseline, Without Survey Weights

Supplement 2.
Data Sharing Statement
