# Supplementary material for: Association of Electronic Cigarette Use by US Adolescents With Subsequent Persistent Cigarette Smoking
Source: JAMA Netw Open. 2023 Mar 27;6(3):e234885. doi: 10.1001/jamanetworkopen.2023.4885 (PMC10043747; doi:10.1001/jamanetworkopen.2023.4885)
Supplement: Supplement 1. — eTable 1. Global Appraisal of Individual Needs–Short Screener (GAIN-SS) Items eTable 2. Association Between Baseline e-Cigarette Use and Continued Cigarette Use (Continued Smoking Measure II) in 3 Years, After Cigarette Initiation a Year After the Baseline eTable 3. Association Between Baseline e-Cigarette Use and Continued Cigarette Use (Continued Smoking Measure III) in 3 Years, After Cigarette Initiation a Year After the Baseline eTable 4. Association Between Baseline e-Cigarette Use and Continued Cigarette Use in 3 Years, After Cigarette Initiation a Year After the Baseline, With Alternative Measures of Continued Use eTable 5. Association Between Baseline Past 12-Month e-Cigarette Use and Continued Cigarette Use in 3 Years, After Cigarette Initiation a Year After the Baseline eTable 6. Association Between Baseline e-Cigarette Use and Continued Cigarette Use in 3 Years, After Cigarette Initiation a Year After the Baseline, Without Survey Weights [file jamanetwopen-e234885-s001.pdf]

Supplemental Online Content

Sun R, Méndez D, Warner KE. Association of electronic cigarette use by US adolescents with subsequent persistent cigarette smoking. *JAMA Netw Open*. 2023;6(3):e234885. doi:10.1001/jamanetworkopen.2023.4885

eTable 1. Global Appraisal of Individual Needs–Short Screener (GAIN-SS) Items
eTable 2. Association Between Baseline e-Cigarette Use and Continued Cigarette Use (Continued Smoking Measure II) in 3 Years, After Cigarette Initiation a Year After the Baseline
eTable 3. Association Between Baseline e-Cigarette Use and Continued Cigarette Use (Continued Smoking Measure III) in 3 Years, After Cigarette Initiation a Year After the Baseline
eTable 4. Association Between Baseline e-Cigarette Use and Continued Cigarette Use in 3 Years, After Cigarette Initiation a Year After the Baseline, With Alternative Measures of Continued Use
eTable 5. Association Between Baseline Past 12-Month e-Cigarette Use and Continued Cigarette Use in 3 Years, After Cigarette Initiation a Year After the Baseline
eTable 6. Association Between Baseline e-Cigarette Use and Continued Cigarette Use in 3 Years, After Cigarette Initiation a Year After the Baseline, Without Survey Weights

This supplemental material has been provided by the authors to give readers additional information about their work.

© 2023 Sun R et al. *JAMA Network Open.*

eTable 1. Global Appraisal of Individual Needs–Short Screener (GAIN-SS) Items

Internalizing Problem Symptoms	1) Feeling very trapped, lonely, sad, blue, depressed or hopeless about the future<br>2) Sleep trouble – such as bad dreams, sleeping restlessly or falling asleep during the day<br>3) Feeling very anxious, nervous, tense, scared, panicked or like something bad was going to happen<br>4) Becoming very distressed and upset when something reminded you of the past
Externalizing Problem Symptoms	1) Lied or conned to get things you wanted or to avoid having to do something<br>2) Had a hard time paying attention at school, work or home<br>3) Had a hard time listening to instructions at school, work or home<br>4) Were a bully or threatened other people<br>5) Started physical fights with other people<br>6) Felt restless or the need to run around or climb on things<br>7) Gave answers before the other person finished asking the question

Notes
Severity of internalizing problem in the past 12 months: 0-1 symptoms (low), 2-3 symptoms (moderate), 4 symptoms (high).
Severity of externalizing problem in the past 12 months: 0-1 symptoms (low), 2-3 symptoms (moderate), ≥4 symptoms (high).
High internalizing problem scores are closely associated with disorders such as depression, anxiety, trauma, and schizophrenia and bipolar disorder. High externalizing problem scores are closely associated with diagnoses of attention deficit disorders and impulsivity.

© 2023 Sun R et al. *JAMA Network Open.*

eTable 2. Association Between Baseline e-Cigarette Use and Continued Cigarette Use (Continued Smoking Measure II) in 3 Years, After Cigarette Initiation a Year After the Baseline

Wave 3 Never Cigarette Users	Continued Cigarette Use Continued Smoking Measure II (CSM-II)[a]	
	aOR (95% CI)	aOR (95% CI)
Ever e-cigarette use	**1.81 (1.03-3.18)**	
Current e-cigarette use		**2.66 (1.07-6.63)**
Age (REF: 12-14)		
15-17	**2.60 (1.58-4.29)**	**2.55 (1.57-4.13)**
Sex (REF: Male)		
Female	0.87 (0.54-1.39)	0.87 (0.54-1.40)
Race/Ethnicity (REF: Non-Hispanic white)		
Hispanic	**0.53 (0.29-0.98)**	0.55 (0.30-1.01)
Non-Hispanic black	**0.10 (0.02-0.65)**	**0.11 (0.02-0.65)**
Non-Hispanic other[b]	0.41 (0.09-1.94)	0.52 (0.13-2.15)
Highest parental education (REF: ≤ High school/ GED)		
Some college	0.98 (0.57-1.67)	1.01 (0.59-1.71)
≥ College	0.76 (0.40-1.43)	0.75 (0.40-1.42)
Household income (REF: <50k)		
50k to 100k	0.80 (0.43-1.47)	0.83 (0.46-1.49)
> 100k	0.79 (0.40-1.54)	0.77 (0.39-1.53)
School grades (REF: < mostly B's)		
≥ mostly B's	0.65 (0.41-1.03)	0.65 (0.41-1.03)
Family tobacco use	**1.77 (1.00-3.12)**	**1.79 (1.02-3.11)**
Secondhand smoke	1.58 (0.87-2.90)	1.52 (0.83-2.79)
Peer cigarette use	1.32 (0.80-2.18)	1.42 (0.89-2.29)
Ever used other tobacco products[c]	1.31 (0.63-2.72)	1.52 (0.75-3.11)
Used alcohol in past 12 months	1.67 (0.96-2.91)	1.70 (0.98-2.96)
Used cannabis in past 12 months	1.49 (0.64-3.46)	1.60 (0.64-4.03)
Susceptible to cigarettes	**3.97 (2.50-6.31)**	**4.07 (2.59-6.40)**
Internalizing problems[d](REF: Low)		
Moderate	1.45 (0.75-2.81)	1.44 (0.74-2.80)
High	1.56 (0.73-3.34)	1.57 (0.73-3.39)
Externalizing problems[d] (REF: Low)		
Moderate	0.80 (0.43-1.48)	0.79 (0.43-1.44)
High	0.97 (0.50-1.87)	0.96 (0.50-1.85)

Notes
Values are bolded if p<.05.
[a] Past 12-month use at wave 4 and past 30-day use at wave 5.
[b] Non-Hispanic other includes American Indian or Alaska Native, Asian Indian, Chinese, Filipino, Japanese, Korean, Vietnamese, other Asian, Native Hawaiian, Guamanian or Chamorro, Samoan, and other Pacific Islander.
[c] Other tobacco products include cigarettes, cigar, pipe, hookah, snus, smokeless tobacco, bidi, kretek, and dissolvable tobacco.
[d] Assessed by the Global Appraisal of Individual Needs-Short Screener (GAIN-SS). Adolescents classified as low (0-1 symptom), moderate (2-3 symptoms), or high (≥ 4 symptoms).

© 2023 Sun R et al. *JAMA Network Open.*

eTable 3. Association Between Baseline e-Cigarette Use and Continued Cigarette Use (Continued Smoking Measure III) in 3 Years, After Cigarette Initiation a Year After the Baseline

**Wave 3 Never Cigarette Users**	**Continued Cigarette Use Continued Smoking Measure III (CSM-III)[a]** aOR (95% CI)	aOR (95% CI)
Ever e-cigarette use	**2.24 (1.06-4.72)**	
Current e-cigarette use		**4.59 (1.39-15.16)**
Age (REF: 12-14)		
15-17	**5.16 (2.00-13.29)**	**4.58 (1.82-11.55)**
Sex (REF: Male)		
Female	0.51 (0.24-1.09)	0.52 (0.25-1.10)
Race/Ethnicity (REF: Non-Hispanic white)		
Other	**0.29 (0.10-0.89)**	**0.34 (0.12-0.94)**
Highest parental education (REF: ≤ High school/ GED)		
Some college	0.61 (0.31-1.21)	0.68 (0.34-1.35)
≥ College	0.71 (0.27-1.87)	0.70 (0.27-1.84)
Household income (REF: <50k)		
50k to 100k	0.98 (0.35-2.74)	1.10 (0.45-2.73)
> 100k	0.76 (0.23-2.52)	0.77 (0.24-2.55)
School grades (REF: < mostly B's)		
≥ mostly B's	0.74 (0.37-1.49)	0.76 (0.40-1.45)
Family tobacco use	2.00 (0.97-4.12)	2.06 (1.00-4.25)
Secondhand smoke	1.88 (0.85-4.15)	1.78 (0.78-4.07)
Peer cigarette use	0.86 (0.40-1.87)	0.97 (0.46-2.07)
Ever used other tobacco products[b]	1.21 (0.46-3.23)	1.43 (0.54-3.76)
Used alcohol in past 12 months	1.36 (0.58-3.16)	1.35 (0.59-3.08)
Used cannabis in past 12 months	1.42 (0.38-5.25)	1.53 (0.39-6.03)
Susceptible to cigarettes	**6.09 (1.83-20.29)**	**6.15 (1.88-20.11)**
Internalizing problems[c] (REF: Low)		
Moderate	0.88 (0.27-2.87)	0.88 (0.27-2.84)
High	2.79 (0.98-7.90)	2.75 (0.96-7.85)
Externalizing problems[c] (REF: Low)		
Moderate	1.22 (0.35-4.30)	1.22 (0.35-4.21)
High	1.22 (0.33-4.49)	1.27 (0.35-4.59)

Notes
Values are bolded if p<.05.
[a] Past 12-month use at wave 4 and established use at wave 5. Established use defined as lifetime ≥ 100 cigarettes and currently smoking.
[b] Other tobacco products include cigarettes, cigar, pipe, hookah, snus, smokeless tobacco, bidi, kretek, and dissolvable tobacco.
[c] Assessed by the Global Appraisal of Individual Needs-Short Screener (GAIN-SS). Adolescents classified as low (0-1 symptom), moderate (2-3 symptoms), or high (≥ 4 symptoms).

© 2023 Sun R et al. *JAMA Network Open.*

eTable 4. Association Between Baseline e-Cigarette Use and Continued Cigarette Use in 3 Years, After Cigarette Initiation a Year After the Baseline, With Alternative Measures of Continued Use

	Wave 5 Continued Cigarette Use			
**Wave 3 E-cigarette Use**	aOR[a] (95% CI)	aRD[b] (95% CI)	Risk without e-cigarette use[c] % (95% CI)	Risk with e-cigarette use[c] % (95% CI)
	Continued Smoking Measure I (CSM-I)[d]			
Ever use	**2.04 (1.28-3.25)**	**1.66 (0.33-2.99)**	1.84 (1.38-2.30)	3.50 (2.17-4.83)
Current use	2.16 (0.99-4.72)	2.02 (-0.74-4.77)	2.09 (1.64-2.54)	4.11 (1.26-6.96)
	Continued Smoking Measure IV (CSM-IV)[e,f]			
Ever use	2.62 (0.87-7.90)	0.41 (-0.37-1.19)	0.27 (-0.08-0.62)	0.68 (-0.31-1.66)
Current use	**7.00 (1.25-39.35)**	1.59 (-0.99-4.16)	0.30 (-0.10-0.70)	1.89 (-0.79-4.57)
	Continued Smoking Measure V (CSM-V)[f,g]			
Ever use	0.80 (0.10-6.49)	-0.03 (-0.33-0.27)	0.17 (-0.30-0.64)	0.13 (-0.31-0.58)
Current use	3.14 (0.13-74.96)	0.31 (-1.36-1.99)	0.15 (-0.27-0.58)	0.47 (-1.46-2.39)

Notes
Values are bolded if p<.05.
[a] Adjusted odds ratio. Adjusted for all study covariates: age, sex, race/ethnicity, highest parental education, household income, school grades, family tobacco use, secondhand smoke, peer cigarette use, ever tobacco product use, past 12-month alcohol use, past 12-month cannabis use, cigarette susceptibility, internalizing problems, and externalizing problems.
[b] Adjusted risk difference, in percentage points, calculated as risk with e-cigarette use minus risk without e-cigarette use. Adjusted for all study covariates, same as listed above.
[c] Estimated risks for continued cigarette use in three years given baseline e-cigarette use status.
[d] Past 12-month use at wave 4 and past 12-month use at wave 5.
[e] Past 12-month use at wave 4 with established use and ≥ 5 days use in the past 30 days at wave 5.
[f] Due to the limited number of non-Hispanic blacks who reported continued use, we replaced the categorical measure of race (non-Hispanic white, non-Hispanic black, Hispanic, and non-Hispanic other) with a binary measure (non-Hispanic white vs other).
[g] Past 12-month use at wave 4 with established use and ≥ 20 days use in the past 30 days at wave 5.

© 2023 Sun R et al. *JAMA Network Open.*

eTable 5. Association Between Baseline Past 12-Month e-Cigarette Use and Continued Cigarette Use in 3 Years, After Cigarette Initiation a Year After the Baseline

	**Wave 5 Continued Cigarette Use**			
**Wave 3 E-cigarette Use**	aOR[a] (95% CI)	aRD[b] (95% CI)	Risk without e-cigarette use[c] % (95% CI)	Risk with e-cigarette use[c] % (95% CI)
	Continued Smoking Measure II (CSM-II)[d]			
Past 12-month use	**2.30 (1.25-4.27)**	**1.39 (0.02-2.76)**	1.18 (0.80-1.57)	2.57 (1.15-3.99)
	Continued Smoking Measure III (CSM-III)[e,f]			
Past 12-month use	**3.21 (1.45-7.10)**	1.04 (-0.08-2.17)	0.53 (0.20-0.85)	1.57 (0.31-2.83)

Notes

Values are bolded if p<.05.

[a] Adjusted odds ratio. Adjusted for all study covariates: age, sex, race/ethnicity, highest parental education, household income, school grades, family tobacco use, secondhand smoke, peer cigarette use, ever other tobacco product use, past 12-month alcohol use, past 12-month cannabis use, cigarette susceptibility, internalizing problems, and externalizing problems.

[b] Adjusted risk difference, in percentage points, calculated as risk with e-cigarette use minus risk without e-cigarette use. Adjusted for all study covariates, same as listed above.

[c] Estimated risks for continued cigarette use in three years given baseline e-cigarette use status.

[d] Past 12-month use at wave 4 and past 30-day use at wave 5.

[e] Past 12-month use at wave 4 and established use at wave 5. Established use defined as lifetime $\geq$ 100 cigarettes and currently smoking.

[f] Due to the limited number of non-Hispanic blacks who reported continued use (past 12-month use at wave 4 and established use at wave 5), we replaced the categorical measure of race (non-Hispanic white, non-Hispanic black, Hispanic, and non-Hispanic other) with a binary measure (non-Hispanic white vs other).

© 2023 Sun R et al. *JAMA Network Open.*

eTable 6. Association Between Baseline e-Cigarette Use and Continued Cigarette Use in 3 Years, After Cigarette Initiation a Year After the Baseline, Without Survey Weights

	**Wave 5 Continued Cigarette Use**			
**Wave 3 E-cigarette Use**	aOR[a] (95% CI)	aRD[b] (95% CI)	Risk without e-cigarette use[c] % (95% CI)	Risk with e-cigarette use[c] % (95% CI)
	Continued Smoking Measure II (CSM-II)[d]			
Ever use	**1.91 (1.12-3.25)**	**0.96 (0.02-1.90)**	1.17 (0.88-1.46)	2.13 (1.26-3.00)
Current use	**2.76 (1.16-6.57)**	1.97 (-0.44-4.37)	1.30 (1.03-1.57)	3.27 (0.87-5.66)
	Continued Smoking Measure III (CSM-III)[e,f]			
Ever use	2.07 (0.94-4.53)	0.46 (-0.13-1.06)	0.48 (0.29-0.67)	0.94 (0.39-1.50)
Current use	**4.70 (1.67-13.28)**	1.62 (-0.18-3.42)	0.53 (0.36-0.71)	2.15 (0.36-3.94)

Notes
Values are bolded if p<.05.
[a] Adjusted odds ratio. Adjusted for all study covariates: age, sex, race/ethnicity, highest parental education, household income, school grades, family tobacco use, secondhand smoke, peer cigarette use, ever other tobacco product use, past 12-month alcohol use, past 12-month cannabis use, cigarette susceptibility, internalizing problems, and externalizing problems.
[b] Adjusted risk difference, in percentage points, calculated as risk with e-cigarette use minus risk without e-cigarette use. Adjusted for all study covariates, same as listed above.
[c] Estimated risks for continued cigarette use in three years given baseline e-cigarette use status.
[d] Past 12-month use at wave 4 and past 30-day use at wave 5.
[e] Past 12-month use at wave 4 and established use at wave 5. Established use defined as lifetime ≥ 100 cigarettes and currently smoking.
[f] Due to the limited number of non-Hispanic blacks who reported continued use (past 12-month use at wave 4 and established use at wave 5), we replaced the categorical measure of race (non-Hispanic white, non-Hispanic black, Hispanic, and non-Hispanic other) with a binary measure (non-Hispanic white vs other).

© 2023 Sun R et al. *JAMA Network Open.*
