# Supplementary material for: Association of Electronic Cigarette Use by US Adolescents With Subsequent Persistent Cigarette Smoking
Source: JAMA Netw Open. 2023 Mar 27;6(3):e234885. doi: 10.1001/jamanetworkopen.2023.4885 (PMC10043747; doi:10.1001/jamanetworkopen.2023.4885)
Supplement: Supplement 2. — Data Sharing Statement [file jamanetwopen-e234885-s002.pdf]

Data Sharing Statement

Sun. Association of Electronic Cigarette Use by US Adolescents With Subsequent Persistent Cigarette Smoking. *JAMA Netw Open*. Published March 27, 2023. doi:10.1001/jamanetworkopen.2023.4885

Data
Data available: Yes
Data types: Deidentified participant data
How to access data: https://www.icpsr.umich.edu/web/NAHDAP/studies/36498
When available: beginning date: 11-22-2022

Supporting Documents
Document types: None

Additional Information
Who can access the data: Anyone having an account with ICPSR can access the data.
Types of analyses: For any purpose.
Mechanisms of data availability: Anyone having an account with ICPSR can access the data.
